# Beyond the bilingualism myth: toward culturally sustaining autism interventions for multilingual families

**DOI:** 10.3389/fpsyt.2026.1767278

**Published:** 2026-03-27

**Authors:** Fernanda A. Castellón, Laura R. Gómez, Tatiana Ramos-Gallardo

**Affiliations:** 1 Department of Counseling, Clinical, and School Psychology, Koegel Autism Center, University of California, Santa Barbara, CA, United States; 2 School of Special Education, School Psychology, and Early Childhood Studies, University of Florida, Gainesville, FL, United States; 3 School of Speech, Language, and Hearing Sciences, San Diego State University, San Diego, CA, United States; 4 Joint Doctoral Program in Language and Communicative Disorders, University of California, San Diego, San Diego, CA, United States

**Keywords:** autism, bilingualism, cultural humility, culturally sustaining practices, heritage language, linguistic hegemony, multilingualism, neuroaffirming interventions

## Abstract

Despite robust evidence that bilingualism does not hinder language development in autistic children, service providers continue advising multilingual families to adopt English as their home language. This research-to-practice gap severs intergenerational cultural transmission and compromises parent-child communication. This perspective paper examines how historical misconceptions linking bilingualism with cognitive deficits became embedded in autism intervention practices. We analyze how intersecting ideologies of ableism and racism co-construct deficit-lens perspectives, and how policies rooted in White Mainstream English hegemony, organizational barriers, and assimilationist paradigms undermine multilingual families’ linguistic practices. Research shows autistic children successfully acquire multiple languages, and heritage language maintenance is essential for ethnic identity, family relationships, and well-being. Yet the systematic exclusion of non-English speakers from intervention research has created an evidence base that reinforces monolingual practices. This disconnect represents a fundamental refusal to dismantle structures positioning English monolingualism as the default standard. Culturally sustaining autism interventions must promote additive bilingual environments, recognizing language as the medium through which children access family histories, spiritual practices, and belonging. Adopting a neuroaffirming framework centering well-being over compliance requires cultural humility and self-reflection about how our experiences shape clinical work. We call for research investigating best practices that honors, protects, and sustains heritage language environments—evidence urgently needed to reshape outdated policies restricting home language instruction.

## Introduction

### Misconceptions about bilingualism and autism

Negative perceptions towards bilingualism can be traced back to early work that found associations between low IQ on a verbal monolingual intelligence assessment and the bilingual experience (1); naturally, the methodology, which erroneously reported causal relationships between bilingualism, emotional maladjustment, “mental confusion,” and “retardation;” has been extensively criticized (2). Early inflammatory claims were refuted by an increase in research on the cognitive implications of bilingualism. To date, researchers and practitioners alike advocate for appropriate assessment practices globally (e.g., selection of bilingually normed standardized assessments, dynamic assessment, and response to intervention) (e.g., 3–5). While evidence for metalinguistic and executive function advantages among neurotypical bilingual children, known today as the bilingual advantage (6–9) has received substantial support, recent studies highlighted an ongoing debate about the robustness and consistency of these cognitive benefits (10–12). These studies suggest the advantage may be task-specific, age-dependent, or moderated by methodological factors (13, 14). Regardless of this ongoing scholarly debate about specific cognitive mechanisms, negative views of bilingualism and autism persist. Early claims of “mental confusion” and ableist views of language acquisition in populations with atypical language development trajectories had already seeped into practice (15) by the time scholars began to study bilingualism and autism (16).

Autism interventions often involve an interdisciplinary team of professionals; therefore, the content and recommendations in this perspective article are relevant to all practitioners who work with multilingual/bilingual (i.e., speakers of two or more languages) autistic children. Throughout this article, we use the term “practitioners” to refer broadly to the education and healthcare professionals, including psychiatrists who oversee care coordination, speech-language pathologists delivering language interventions, occupational therapists supporting sensory and motor development, behavior analysts who design and implement behavioral interventions, early interventionists who support young autistic children, and educators working with autistic children in school settings. Similarly, we will use *multilingual/multilingualism* throughout the paper to include experiences of individuals who speak or sign more than one language.

Misinformation around multilingualism and autism has proved to be a significant barrier to multilingual caregivers and their autistic children; indeed, recommendations to speak the societal language (SL) uniquely within the home environment have prevented families from accessing and providing the full range of heritage language (HL) exposure thereby, limiting language development opportunities (e.g., 17). In children without dis/abilities, multilingual language exposure has been associated with the following: vocabulary growth across languages (18), family communication (19), and racial-ethnic identity (20). Furthermore, in a study conducted with bilingual autistic children, language exposure (i.e., the proportion of opportunities to access the HL and SL) was the greatest predictor of language proficiency; moreover, they found that balanced bilingual children 1) performed similarly to monolingual peers on vocabulary, grammar, and story cohesion measures, and 2) performed better on a pragmatic task (21). These findings directly contradict pervasive myths about bilingual language development in autistic children and highlight the importance of HL exposure and potential advantages associated with bilingualism (i.e., pragmatics).

Intersecting ideologies (i.e., ableism and racism) co-construct negative beliefs and values related to language and learning abilities in multilingual children with dis/abilities that influence clinical deficit-lens perspectives informing practice (22, 23). In communities that perpetuate majority language hegemonic values to maintain power over minoritized communities, caregivers are often told to adopt the majority language as their home language; in the context of the United States, societally-imposed ideas and values promote White Mainstream English dominance at the cost of heritage languages (e.g., Spanish, Black Language^1^, etc.) (24). Research documents how caregivers are pushed to adopt English as their home language because service providers tell them that providing multilingual exposure will result in further language delays for their child (26, 27). The multilingualism myth in the autism field has been inadvertently fed by the systematic exclusion of non-English-speaking participants in intervention studies (28), failing to provide evidence for language strategies delivered in a language other than English. Practitioners following these evidence-based practices are forced to endorse English-only practices and have the unintended consequence of damaging parent-child communication channels (17, 29).^1^

### Research-to-practice gap in interventions for multilingual autistic children

Recent research on bilingualism and autism suggests that bilingual exposure does not negatively impact language development in children with autism (15). Instead, it may offer cognitive, social, and language benefits (30–32). Studies indicate that autistic children can successfully acquire multiple languages with developmental trajectories similar to those of monolingual autistic peers, including delayed language development when compared to neurotypical peers, echolalia, and social communication difficulties (33, 34). These findings support the notion that bilingual families should not be discouraged from using their home language. Instead, interventions should be designed to support the families’ languages, recognizing the cultural and social importance of multilingualism. These practices characterize the components of a culturally sustaining framework, focused on not only integrating but also preserving the child’s and the family’s home language and cultural traditions (35). Culturally sustaining practices actively seek to preserve the child’s and the family’s identity by fostering the continuation of diverse cultural practices (36). However, some studies have revealed variability regarding practitioners’ recommendations, with some professionals actively supporting bilingualism while others persist in advising against multilingual exposure (26, 37, 38). Moreover, some caregivers reported believing that exposure to two languages would generate or further exacerbate language-learning difficulties in their children; this belief was reinforced by professionals’ recommendations to use English only at home (39, 40). This persistent gap between evidence and practice reveals how deeply English monolingualism remains embedded in autism service delivery systems.

### Current practices in the United States

#### Education policy

Persistent ignorance of the evidence supporting promotion of home languages within intervention and assessment suggests that these language recommendations are systematically motivated by external system-level beliefs and practices. Systemic implicit and explicit inculcation of majority language (i.e., English) hegemony shapes education, intervention, and healthcare policies (41). These policies restrict home language instruction and promote English-only language policies, which have lasting impacts at the community (e.g., administrative) and individual (e.g., practitioner and family) levels. Studies have found that historical education policy motivated by subtractive views of bilingualism (e.g., Proposition 227 in California/United States, and the “First Four Hour” policy in the Northern Territory/Australia) has fostered persistent negative attitudes towards bilingualism and bilingual education (41–43). The consequences of this infrastructure are profound for multilingual autistic students. Despite improved global policy supporting additive bilingual pedagogy, a critical gap between research and practice remains (44). Providers servicing multilingual families report positive views towards bilingualism, but also report several barriers impacting active support of heritage languages within interventions (e.g., lack of confidence in advice, confusion surrounding the most recent research/best practice, increased reluctance associated with students’ language abilities and support needs). This research-to-practice gap helps explain persistent clinical English language recommendations documented globally (27, 45, 46).

Multilingual students with disabilities, including those with autism, face compounded educational barriers when English-only policies intersect with special education placement decisions. Educational pressure to develop English proficiency among multilingual learners with disabilities results in higher rates of alternative curriculum placement (47, 48). Special education placement determinations disproportionately place autistic students of color within restrictive settings that implement a modified curriculum (e.g., increased proportion of autistic students of color in the United States participating in the general education setting less than 80% of their day; 49). Specifically, decisions to utilize a modified curriculum directly track students into pathways receiving modified alternative diplomas, thereby negatively affecting the probability of academic achievement, access to postsecondary education, and future employment opportunities. Research has shown that multilingual learners are often overidentified for special education services (49–51) and when language barriers are misinterpreted as a disability, students may be placed into inappropriate educational settings that prioritize English acquisition over maintaining bilingual development (50, 52). While we have discussed trends in the United States, similar disproportionate identification patterns have been documented in other countries (e.g., United Kingdom, Australia, New Zealand, and Sweden; 53).

#### Practitioners

Practitioners who provide autism services to multilingual families navigate complex professional landscapes, often shaped by their own cultural and linguistic backgrounds, organizational constraints, and systemic inequities (e.g., administrative support, company policies, tokenization, etc.). Understanding these experiences is critical for advancing equity in autism research and practice, particularly as the literature on practitioners’ experiences and challenges seems to be an emerging field in research (37, 54, 55). There is some evidence highlighting practitioners’ challenges, particularly for those working with families living in poverty, complex family dynamics, and ethnic minority families who report that coaching families who primarily spoke languages other than English was perceived as interfering with coaching effectiveness (56). Currently, there is a lack of diverse, multilingual practitioners providing services to autistic children and their families (55, 57). Practitioners who share a similar marginalized identity and linguistic background with families report improved relationships with caregivers, including communication and connection, and positive clinical outcomes for children (58). However, these positive experiences also come with challenges and needs, such as the lack of translated resources, mentorship, and training (55, 59). In addition to this, bilingual practitioners report an additional workload that is not compensated, due to assumptions about their ability to translate materials as dual-language speakers (37, 54, 55). For practitioners who are monolingual, there is a barrier to communicating and establishing a relationship with families, qualified interpreters are often inaccessible, and when available, they often lack discipline-specific terminology that affects the communication between the practitioner and the family (37).

Evidence that practitioners recommend avoiding heritage language use shows the urgent need for updated evidence-based training in best practices for multilingual, autistic children (60). However, practitioners, including those who recognize the value of bilingualism and wish to support families’ home language, find themselves in complicated situations when organizational and system-level policies do not support culturally sustaining practices for multilingual families and children (29, 54, 61, 62). There is a notable tension between practitioners' experiences and systematic constraints, as practitioners' beliefs at the individual level do not always align with their actual recommendations or service delivery (29). While practitioners express strong support for multilingualism in principle, they simultaneously participate—sometimes unwillingly—in institutional practices that undermine multilingual care, such as overburdening the sole bilingual staff member or declining to serve families who speak languages other than English (29). How organizations structure their policies and operations substantially influences practitioners’ ability and willingness to implement culturally and linguistically responsive adaptations for multilingual families (54). Critical organizational barriers that impede culturally sustaining multilingual practices include inadequate professional development systems, the absence of structured mentorship programs that build cultural and linguistic competence, the recruitment of diverse staff without corresponding institutional support for their increased responsibilities, and inflexible service delivery policies that fail to accommodate families’ cultural and linguistic needs (54).

#### Families

Families often accept clinical English language recommendations, which exacerbate existing concerns about their children’s future well-being and academic success (40, 45, 63). Fueled by linguistic hegemony in the United States, White Mainstream English (WME) is positioned not only as the only academic language but also as a marker of intelligence, competency, and future success (22, 24). In this way, promotion of WME is a strategy for maintaining cultural, economic, and political dominance over minoritized communities. Fear of further oppression and aggravated “otherness” (23) critically informs families’ clinical interactions and inclination to follow English-language recommendations.

Bilingual families of autistic children positively regard heritage language knowledge, reporting that heritage language maintenance is essential for participation in family and community-related activities (e.g., religious activities, family reunions, cultural events, and future work opportunities; 46, 64). Therefore, pervasive early-intervention English language recommendations are in direct opposition to family values, perspectives, and goals for their children. Moreover, these recommendations are extremely costly for heritage language maintenance as they can aggravate existing systemic pressures to adhere to the majority language (e.g., English) and culture (i.e., acculturate). Bilingual families of young children receiving special education services report feeling more comfortable and effective when using their home languages (65), and increased tension and additional stress due to clinical English language recommendations (39, 40, 66). These recommendations potentially compromise the quality of parent-child interactions, which are integral to the intervention program and language development (67). Recommendations to only speak English with bilingual children deprive them of forming meaningful memories, bonds, and community ties that contribute significantly to a future sense of belonging and identity.

Decreased heritage language proficiency is associated with weaker ties with heritage culture (68). Moreover, not only is heritage language proficiency a predictor of ethnic identity, but it is also associated with the quality of parent-child relationships (20). Deprivation of continued growth and maintenance of children’s heritage language additionally limits families’ shared language and significantly hinders parent-child communication (19, 69). Ethnic-racial identity among Latine youth is associated with important markers of well-being. For example, stronger ethnic-racial identity is associated with higher self-esteem among Latine youth (70). Without access to essential protective factors that promote known predictors of well-being (e.g., cultural identity, heritage language, community access), families are at increased risk for aggravated acculturation stress and negative well-being outcomes.

## Discussion

Unanimously across disciplines, languages, and cultural contexts, research demonstrates that bilingualism does not hinder language development in autistic children (15, 30–32). Yet this evidence remains siloed in academic journals, failing to reach the practitioners who continue to advise families to abandon their heritage languages (26, 37, 38). This disconnect represents not only a knowledge translation gap but a fundamental failure to dismantle the structures that position English monolingualism as the default standard of progress.

Culturally sustaining autism interventions would instead promote additive bilingual environments, harnessing the strengths of caregivers’ and the family’s linguistic ecology. Such approaches recognize that language is the medium through which children access their family histories, spiritual practices, humor, affection, and belonging. For example, practitioners who engage in culturally sustaining practices would go beyond “adapting” interventions, intentionally looking for different ways to preserve the family’s heritage (36). This would include providing services in the family’s preferred language when possible, and for monolingual practitioners, establishing collaborative relationships with bilingual colleagues, interpreters, and cultural brokers. The collaborative relationship could result in creating personalized books that include words and images about the child’s world and languages, building directly on their experiences (71). It may even expand to creating social stories and role-plays that reflect the child’s actual cultural experiences and community contexts. These types of practices place the child’s and the family’s culture and language as the source of expertise that shapes intervention goals, methods, and outcomes. When we strip autistic children of their heritage languages, we sever intergenerational transmission of culture and fracture the parent-child relationship at its most intimate level. Mothers who cannot share lullabies in their first language, grandparents who become linguistic strangers to their grandchildren—these are not unfortunate side effects of intervention, but predictable consequences of assimilationist practices masquerading as clinical best practice.

A neuroaffirming approach- one that positions autism as a natural form of human neurodiversity rather than a disorder requiring normalization (72, 73) and adopts strength-based perspectives that honor autistic ways of communication and interaction (74)- demands that we recenter well-being as the primary metric of intervention success, rather than compliance or assimilation. Well-being for autistic children from multilingual homes cannot be separated from their ability to access their full linguistic repertoire, maintain meaningful relationships with nuclear and extended family members, and develop positive identities that embrace neurodivergence, cultural heritage, and their sense of belonging to a community. Current intervention paradigms measure success through tools normed on White, English-speaking, middle-class populations—instruments not intended to capture the quality-of-life factors relevant to bi/multilingual, multicultural autistic individuals.

Moving towards autism interventions that promote cultural sustainability and multilingualism as foundational principles has the potential to reduce the stigma surrounding autism in minority communities. Instead of positioning autism as incompatible with heritage language maintenance, practitioners can enact cultural change alongside families. Critical Autism Studies (75, p. 1), which calls for “advancing enabling narratives of autism to challenge deficit-focused constructions,” presents a framework centering autistic culture, calling us to conceptualize multiculturalism with both neurodivergence and ethnic belonging.

### Implications: research and practice

Regardless of your professional role, whether you are a psychiatrist, psychologist, speech-language pathologist, behavior analyst, occupational therapist, or educator, it is essential to ask yourself: how have I been working with autistic multilingual children and their families? Engage in self-reflection about your own practice (e.g., research, educational, and clinical): When you recommend intervention services, do you inquire about home language practices, or do you default to English-only practices because these are familiar, convenient, or “evidence-based”? When families express their desire for maintaining their heritage language, do you reassure them with research supporting bilingualism, or do you implicitly communicate—through hesitation or silence—that their fears about bilingualism are justified? Engaging in intentional self-reflection about the influence of our own culture is the first step to understanding the impact it has on our work with these families. Recognizing that our individual experiences shape our worldview, and accepting that others’ experiences shape theirs, is the essence of cultural humility- a disposition that should guide all early intervention services (76).

Concurrently, to support the aforementioned culturally sustaining clinical efforts and to empower practitioners with the tools and evidence necessary to combat the detrimental, pervasive nature of English-only recommendations, researchers must intentionally engage in work that addresses and documents the language and developmental trajectories of bi/multilingual autistic learners. The current research investigating bi/multilingualism in autistic youth falls into two categories thus far: 1) evidence that bi/multilingualism is not harmful to overall language development, and 2) evidence of the harmful effects on parent-child relationships and well-being when heritage languages are not supported. These categories do not yet honor heritage language developmental trajectories, nor do they investigate quality care for autistic youth through culturally sustaining interventions. We have the responsibility to create resources and models for service providers and other practitioners to appropriately support bi/multilingual autistic children. In an increasingly globalized and pluralistic society, representation of diverse linguistic experiences is critical. We therefore encourage the field to recognize and represent multilingual experiences (i.e., exposure to two or more languages or dialects). Herein, we propose creating a third category of investigative inquiry: 3) evidence for best practices honoring, protecting, and sustaining heritage language environments to improve family cohesion and well-being. This line of research is urgently needed to reshape outdated policies restricting home language instruction in education and healthcare.

## Data Availability

The original contributions presented in the study are included in the article/supplementary material. Further inquiries can be directed to the corresponding author.

## Footnotes

1 Black language highlights common language practices, with distinct semantic, syntactic, morphological, and phonological patterns in the Black community in the United States (e.g., African American English and Ebonics) (24, 25).
